# Development and Impact of a Community-Delivered, Multisectoral Lifestyle Management Service for People Living With Type 2 Diabetes (Logan Healthy Living): Protocol for a Pragmatic, Single-Arm Intervention Study

**DOI:** 10.2196/69477

**Published:** 2025-03-10

**Authors:** Sjaan R Gomersall, Denis Y Giguere, Jacqueline Cotugno, Joanna Munro, Wallis J Westbrook, Robyn Littlewood, John Cairney, Elisabeth AH Winkler, Phillip M van der Vliet, Ana D Goode, Tahlia Alsop, Genevieve Nissa Healy

**Affiliations:** 1 Health and Wellbeing Centre for Research Innovation School of Human Movement and Nutrition Sciences The University of Queensland St Lucia Australia; 2 Logan Healthy Living UQ Health Care Ltd Logan Australia; 3 Health and Wellbeing Queensland Milton Australia; 4 UQ Health Care Ltd Toowong Australia

**Keywords:** type 2 diabetes, lifestyle management, allied health, evaluation, protocol

## Abstract

**Background:**

Type 2 diabetes is the fastest-growing chronic condition in Australia, with higher prevalence in disadvantaged groups. Logan Healthy Living by UQ Health Care is a proof-of-concept, interprofessional allied health clinic focused on supporting people with and at risk of type 2 diabetes in Logan, a region in South East Queensland, Australia, with high levels of health inequity. Logan Healthy Living is supported by the Queensland Government through Health and Wellbeing Queensland and a broader multisectoral alliance including primary health care; tertiary hospital and health services; and government, community, and university sectors.

**Objective:**

This paper describes the establishment of Logan Healthy Living and outlines the evaluation protocol for the service’s type 2 diabetes lifestyle management program.

**Methods:**

The context and setting of Logan Healthy Living are presented, and the process for establishing the multisectoral partnerships, development and governance of the service, and the facility are described. The lifestyle management program is an 8-week, group-based program that includes 1 hour of education and 1 hour of supervised, individually tailored exercise each week. The theoretical underpinnings and the program are described in detail. The Reach, Effectiveness, Adoption, Implementation, and Maintenance (RE-AIM) framework will guide evaluation of the program and inform key questions regarding the number and characteristics of the clients (reach); diabetes-related distress, health behaviors (physical activity and diet), quality of life, self-management self-efficacy, loneliness, community involvement, anthropometric measures, hemoglobin A_1c_ levels, physical function, and health care use (effectiveness); referral pathways (adoption); fidelity, appropriateness, acceptability, and costs (implementation); and long-term effectiveness (maintenance). Data will be drawn from a purposefully embedded minimum dataset and data registry, with the process for designing and embedding data collection into practice (via surveys, in-person measures, and client management software) described in detail.

**Results:**

Ethics approval has been obtained for the data registry. Logan Healthy Living is a 4-year proof of concept that concludes on December 31, 2024, with findings expected to be reported starting in 2025.

**Conclusions:**

While multisectoral responses are needed for complex community health challenges, the processes for achieving these are rarely documented, and the description of the development of Logan Healthy Living has the potential to inform future partnerships. The findings of the evaluation will provide important new knowledge on the impact of a community-delivered type 2 diabetes program on individuals, the community, and the health system in an area of high health inequity.

**International Registered Report Identifier (IRRID):**

DERR1-10.2196/69477

## Introduction

### Background

Type 2 diabetes is a chronic condition where the body becomes resistant to the normal effects of insulin and gradually loses the capacity to produce enough insulin in the pancreas [1]. The onset of type 2 diabetes is associated with both nonmodifiable (eg, genetic) and modifiable (eg, health and behavioral) risk factors [1]. Worldwide, in 2020, type 2 diabetes was the ninth leading cause of death, affecting 7% of the population, with prevalence rates only expected to increase [2]. In Australia, type 2 diabetes impacts an estimated 1.2 million people [1], with a further 2 million having prediabetes [2]. It is the fastest-growing chronic condition in Australia [1], with health system expenditure estimated at Aus $3.4 billion (US $2.1 billion) per year [3]. Type 2 diabetes has a significant impact on individuals. Along with the symptoms of the disease itself, many people experience diabetes-related complications (eg, retinopathy, peripheral vascular disease, and ischemic heart disease) and are at increased risk of multimorbidity (eg, cardiometabolic, vascular, and mental health conditions), which collectively impact their quality of life, impair functioning, and increase financial and economic burden [4]. Type 2 diabetes disproportionately affects disadvantaged groups. For example, people of a low socioeconomic status and with low levels of education [5], immigrants, and those from culturally and linguistically diverse backgrounds have increased risk of type 2 diabetes [6]. Furthermore, the intersection of disadvantage is likely to magnify risk and burden of disease [7].

Management of type 2 diabetes is primarily focused on achieving glycemic control through modification of health behaviors (typically physical activity and nutrition) and, where required, medication. Evidence-based management of type 2 diabetes emphasizes person-centered, team-based care with integrated long-term treatment approaches, as well as the involvement of social community support [8,9]. Self-management, where the person with type 2 diabetes works in partnership with their social supports and health professionals to understand, manage, and optimize their health, is the goal. Management of modifiable risk factors is a core component of self-management, with such strategies focused on building positive health behaviors, including physical activity and nutrition, and optimizing psychological well-being [9,10]. Multidisciplinary, group-based approaches to lifestyle modification have consistently been shown to positively impact a range of health and well-being outcomes for people living with type 2 diabetes [11]. To support this in practice, the Australian National Diabetes Strategy (2021-2030) has called for a multisectoral response by government and communities to provide the integrated care required [12].

UQ Health Care (a not-for-profit health care wholly owned enterprise of The University of Queensland) has established Logan Healthy Living as a specialist clinic designed to respond to this need [13]. Purposefully established in South East Queensland in the City of Logan, an area of Queensland with high burden of type 2 diabetes and intersectional disadvantage, Logan Healthy Living was designed with the aim of reducing the burden of disease on individuals, the community, and the health system [13]. The core service of Logan Healthy Living is a group-based lifestyle management program delivered by an interprofessional allied health team focusing on supporting people living with and at risk of type 2 diabetes in Logan. The program has 3 main foci: physical activity, healthy eating, and well-being [14]. Logan Healthy Living is a 4-year proof of concept delivered by a Queensland-first alliance among primary health care; tertiary hospital and health services; and government, community, and university sectors.

A key feature of the establishment of Logan Healthy Living was the integration of an outcome-based funding model—a model that prioritizes value-based care and patient-centered outcomes and leverages multisectoral partnerships. Critical to value-based care models is the collection and use of data to continually evaluate the impact of the service on the participants as well as the health system more broadly [15]. Moreover, leveraging this approach for practice-based evidence generated in “real-world” settings can add to the currently limited evidence on community-based, self-management programs for people living with or at risk of type 2 diabetes [16]. With dimensions at both the individual level and multiple ecological levels, the Reach, Effectiveness, Adoption, Implementation, and Maintenance (RE-AIM) framework [17,18] offers a balanced and pragmatic approach to the evaluation of applied community-delivered programs considering factors such as the characteristics of the participants who take up the program (reach) and participant satisfaction (implementation). The RE-AIM framework has been applied across multiple evaluations, including diabetes health coaching [19], diabetes prevention programs [20], and telephone-delivered type 2 diabetes support [21]. To simultaneously support reporting for the outcome-based funding model and evaluate the real-world impact of a routinely delivered community program, a comprehensive continuous evaluation protocol informed by the RE-AIM framework was developed.

### Objectives

The purpose of this paper is to describe the development of Logan Healthy Living and the protocol for program evaluation of this allied health care service. Specifically, we describe the context and setting of the service, the multisectoral partnership and co-design activities contributing to the service development, governance, and participants and recruitment channels. We then describe the protocol for the evaluation of the service according to the RE-AIM framework.

## Methods

This protocol was prepared using the SPIRIT (Standard Protocol Items: Recommendations for Interventional Trials) checklist [22].

### Context and Setting

Logan Healthy Living by UQ Health Care is located in the City of Logan in South East Queensland, Australia. With an estimated population size of 345,098 [23], the City of Logan is identified as one of the most culturally and linguistically diverse and socioeconomically disadvantaged areas in Australia. The proportions of the population speaking a language other than English at home; being born overseas; identifying as Aboriginal or Torres Strait Islander, Māori, or Samoan; being unemployed; and having one or more chronic health conditions are above South East Queensland averages [23]. The region has also experienced rapid population growth and has an aging population [24]. The City of Logan has a strong focus on community services and fostering connection, with a wide range of no- and low-cost community social programs, including Logan City Council Libraries; arts, culture, and heritage initiatives; sport and recreation facilities (including the Active and Healthy program offering >100 weekly free and low-cost health and well-being activities); and parks and community gardens. Logan also has a diverse range of cultural groups and grassroots networks supporting community connection [25].

In Logan, estimates of the prevalence of type 2 diabetes and associated indicators of burden of disease are consistently higher than state-based estimates for Queensland. Almost 20% (18.5%) of people aged ≥75 years living in Logan have type 2 diabetes (12.4% for those aged 55-74 years and 1.7% for those aged 18-54 years) [26], with the third highest rate of insulin-treated type 2 diabetes in Australia (an indicator of advanced or nonresponsive progression of the disease) [24]. Type 2 diabetes accounts for 31% of potentially preventable hospitalizations in Logan, making it the leading cause of potentially preventable hospitalizations [24]. Those living with type 2 diabetes in the area are also disproportionately likely to die from diabetes-related causes, with the average diabetes-specific mortality rate in Logan being 32% higher than that for Queensland in general [24].

### Development of Logan Healthy Living

The concept of a multisectoral, allied health–delivered lifestyle management program for people living with type 2 diabetes in Logan emerged from an existing partnership between UQ Health Care and Metro South Health (the region’s public health care provider) [27]. In 2014, Metro South Health and UQ Health Care established an innovative, integrated primary-specialist model of care for the medical management of people with complex diabetes, the “Beacon” model [28]. The Beacon model saw complex diabetes management provided within a community practice by a multidisciplinary team consisting of an endocrinologist from Logan Hospital, 2 to 3 general practitioners (GPs) with a special interest in diabetes from UQ Health Care, and a diabetes nurse educator from the Metro South Health community team who provided 1:1 consultation (at times before the GP with a special interest in diabetes to assist in triage). The aim of the Beacon model was to build capacity in primary care for managing complex diabetes through advanced management plans and subsequently diverting people with type 2 diabetes from tertiary care. While the Beacon model demonstrated favorable outcomes in changes in hemoglobin A_1c_ (HbA_1c_) concentration and the percentage of patients meeting combined clinical targets of HbA_1c_ concentration, blood pressure, and low-density lipoprotein cholesterol [29], key stakeholders acknowledged that the Beacon model alone was not sufficient to address the rising community need. Moreover, it lacked a health behavior change program focused on self-management—a critical component for the management of type 2 diabetes [11].

To address these needs, in approximately 2019, what would eventually be known as “Logan Healthy Living” was envisioned, expanding on the existing partnership between Metro South Health and UQ Health Care and integrating learnings from the Beacon model and UQ Health Care’s experience in delivering interprofessional, allied health services at other sites. The first phase of service development was establishing an alliance of partners underpinned by a commitment to prevent chronic disease and keep people well and out of hospital in Logan. UQ Health Care and Metro South Health led the establishment of the alliance, which in turn also included the Brisbane South Primary Health Network, The University of Queensland, and Griffith University. By early 2020, the alliance had established a proof-of-concept model for a comprehensive lifestyle management program delivered by an interprofessional team of allied health professionals and infused with a student allied health workforce.

Concurrently, in 2019, the Queensland Government established the state’s first prevention agency, Health and Wellbeing Queensland [30]. The concept of Logan Healthy Living, a service delivering comprehensive lifestyle management programs to support behavior change and self-management, was put to the board of Health and Wellbeing Queensland by UQ Health Care and Metro South Health on behalf of the alliance. The proposal was strongly aligned with the vision and strategic plan of the newly formed Health and Wellbeing Queensland, which included developing and trialing new models of care for the prevention and management of chronic disease. Here, the intention was to reduce pressure on the tertiary health care system, address health inequities, and build multisectoral partnerships that drive system-level change for improved health and well-being outcomes. In 2021, Health and Wellbeing Queensland joined the alliance, with UQ Health Care and Health and Wellbeing Queensland entering into a 4-year agreement (2021-2024) to support the delivery of a tailored lifestyle management program. UQ Health Care secured additional revenue agreements and financial contributors to deliver on the commitment to reduce financial barriers for participants. The UQ Health Care and Health and Wellbeing Queensland service-level agreement outlined a comprehensive suite of deliverables and biannual key performance indicators. These key performance indicators were designed to evaluate the program according to the RE-AIM indicators across the 3 priorities identified previously (reduce the burden of disease on individuals, the community, and the health system). Examples of the agreed upon key performance indicators are summarized in Table 1.

**Table 1:** Examples of key performance indicators for Logan Healthy Living.

Evaluation domain	Key performance indicator
Reach	Participant’s type 2 diabetes status (at risk, newly diagnosed, complex, or chronic) Participant’s demographic characteristics Participant retention Participant attrition
Effectiveness	Participant’s knowledge, health literacy, and intention to change Participant’s healthy behavior action (health behavior change and anthropometric measures) Health system impact
Adoption	Referral pathways
Implementation	Participant satisfaction Staff satisfaction Participant safety (adverse events) Student training
Maintenance	Participant sustained healthy behavior Sustainable funding

The development and design of the service was community led and iteratively co-designed with key stakeholders, including consumers. Activities included participant journey mapping to inform program and resource development, consultation with student placement providers, and learning needs assessments for local GPs. A key event for consumer and stakeholder engagement was a half-day “design-jam” led by UQ Ventures and held on campus at Griffith University in Meadowbrook, where approximately 60 end users, delivery providers, academics, clinicians, community leaders, and representatives of other key stakeholders (eg, Metro South Health, Brisbane South Primary Health Network, and the Aboriginal and Torres Strait Islander Community Health Service) came together to progress their combined vision and implementation plan for the service. Subsequent planning days have also been hosted, which have similarly included Logan Healthy Living staff, key stakeholders, and consumers.

Simultaneously, the plan for routine collection of reporting and evaluation data was co-designed by key stakeholders, including researchers from The University of Queensland; the Logan Healthy Living Clinical and Operations Manager, administration team, and clinicians; and relevant content experts. Key to successfully collecting the range and breadth of data required was the intent to embed data collection into routine service delivery. Led by a researcher employed by the Health and Wellbeing Centre for Research Innovation (a research center jointly funded by The University of Queensland and Health and Wellbeing Queensland; SG), along with a senior academic experienced in evaluation (GH) and a data analyst (EW), a pragmatic data collection and consent process was co-designed and embedded into daily operations. Data are collected through both practice management software (Gensolve) and clinical trial software (REDCap [Research Electronic Data Capture]; Vanderbilt University) and facilitated day to day by the Logan Healthy Living clinical team, with technical and content support on an as-needed basis from The University of Queensland. Implementation of the data collection process was supported by on-site training sessions and a comprehensive manual developed by the data analyst. The Clinic and Operations Managers have been essential in establishing and building a culture for collaboration and prioritization of data collection.

### Facilities

Logan Healthy Living went live for operations in July 2021. The service initially operated from Griffith University Logan Healthcare Centre, where it was intentionally colocated with the Beacon Clinic and Metro South Health Chronic Disease Diabetes Service (which included diabetes educators, nurse practitioners, and a podiatry service). In February 2023, Logan Healthy Living moved into its permanent, purpose-built location in a dedicated health and medical clinic, Meadowbrook Medical Centre, colocated with the UQ Health Care Meadowbrook GP clinic and the Logan Hospital endocrinology outpatient services (Logan Endocrine and Diabetes Service). The Meadowbrook Medical Centre is centrally located in the City of Logan and positioned within the community’s transport and retail hub, Logan Hospital, and the Logan Healthy Kids Club and Good Start programs operated by Children’s Health Queensland. The site is also set to become part of a larger health precinct, with a planned staged expansion of Logan Hospital and an additional 2 private hospitals.

Logan Healthy Living occupies 400 m^2^ at Meadowbrook Medical Centre and includes a gym floor, 5 curtained consultation spaces, 2 individual consultation rooms, 2 large private rooms (used for staff or student rooms or for group education sessions), and an integrated open space dedicated to delivering group education. The open gym includes a range of exercise equipment, including cardiovascular training equipment (treadmill and stationary bicycle), parallel bars, steps and stairs, and resistance and balance training equipment. UQ Health Care invested in state-of-the-art resistance training machines by HUR Australia [31] that use air resistance and SmartTouch software for automatically programming and tracking load and equipment position (eg, seat heights), with participants tapping on and off the equipment with a personalized radio-frequency identification wristband. HUR Australia equipment was selected to reduce the barriers and improve safety with resistance training for older adults and new exercisers; the HUR Australia equipment is fully automated and applies smooth and consistent resistance across the range of motion.

The clinic is staffed by a range of allied health professionals, including physiotherapy, exercise physiology, dietetics, diabetes education, and health psychology professionals. With its student-infused model, the service also provides clinical education and placements for students from Griffith University and The University of Queensland. These include clinical placements for physiotherapy, exercise physiology, dietetics, and psychology students (ranging from 4 to 20 weeks in length) and project placements in a wide range of health disciplines such as nutrition and dietetics, pharmacy, social work, and health service management. Students from a range of disciplines work together to provide an interprofessional model of care. Beyond providing health care services, Logan Healthy Living is also further embedding itself into the community by contributing to the development and career selection of a locally based workforce, with the aim to promote workforce sustainability in the region. Examples of these activities include facility tours with question-and-answer sessions and opportunities for work experience with the Logan Healthy Living team for local high school students with interest in health careers.

### Governance

Logan Healthy Living is governed by a multisectoral steering committee that is cochaired by UQ Health Care and Health and Wellbeing Queensland. Beyond UQ Health Care and Health and Wellbeing Queensland, the steering committee includes representatives from The University of Queensland, Griffith University, Metro South Health, Logan Hospital, Brisbane South Primary Health Network, and Logan Healthy Living participants. Members of the steering committee are responsible for providing program leadership and direction (eg, understand strategic implications and outcomes and accept responsibility for program strategy and overall benefit realization) and governance (eg, risk identification and mitigation, stakeholder management, and monitoring of progress) and maximizing program benefits (eg, monitor program outputs and monitor implementation and evaluation). Additional stakeholders are also consulted by the steering committee, such as broader consumer groups, the Aboriginal and Torres Strait Islander Community Health Service, Children’s Health Queensland, and Diabetes Australia.

**Figure 1:**
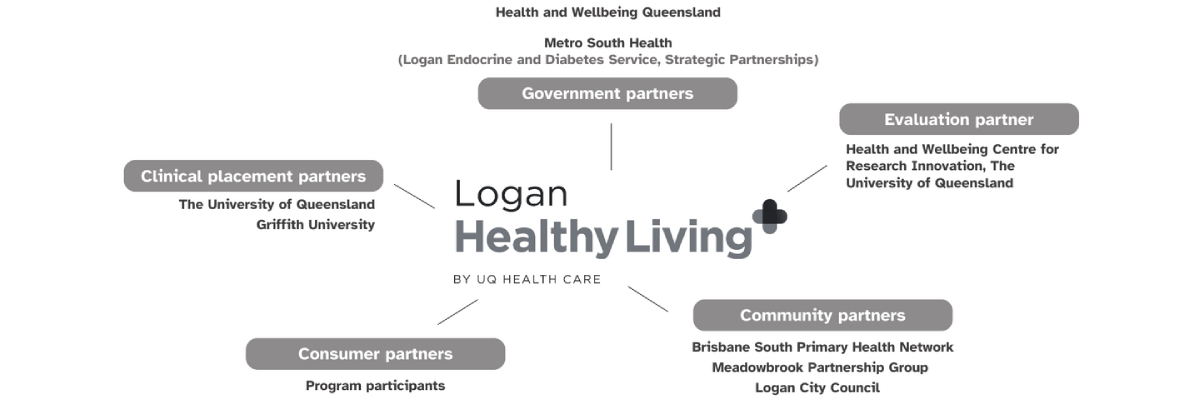
Logan Healthy Living key stakeholder network.

Logan Healthy Living is connected to the broader community governance of the Logan region through the Meadowbrook Partnership Group. Established in 2018, the group comprises representatives from key organizations who are positioned to influence integration and impact in Logan. Membership includes Brisbane South Primary Health Network; Logan City Council; Metro South Health; Economic Development Queensland; Loganlea State High School; The University of Queensland; Griffith University; and the Department of State Development, Infrastructure, Local Government, and Planning. Collectively, the membership represents state, federal, primary care, education, and local government stakeholders. All members have strong community relationships and understanding of local issues. Logan Healthy Living (clinical and operations manager) and UQ Health Care (chief executive officer) are members of the Meadowbrook Partnership Group. Figure 1 shows the network of key stakeholders involved in the development and delivery of Logan Healthy Living.

### Program Cost, Participants, Referral Pathways, and Intake Process

Logan Healthy Living delivers services at no cost to participants. Where possible, reimbursement for the lifestyle management program is sought through Medicare, Australia’s universal health insurance scheme that provides free or subsidized health care (Medicare Benefits Schedule) and selected medication (Pharmaceutical Benefits Scheme, including diabetes medications) for Australian and New Zealand citizens, permanent residents, and some temporary residents. Specifically, Logan Healthy Living seeks reimbursement through the Medicare Benefits Schedule (group allied health service for type 2 diabetes), which funds 1 intake assessment (Aus $74.80 [US $46.99]) and 8 group sessions (Aus $18.65 [US $11.72] per session per person) per calendar year [32]. To claim Medicare benefits, the group sessions must include between 2 and 12 people; last at least 60 minutes; and be delivered by a credentialed diabetes educator, accredited exercise physiologists, or accredited practicing dietitian [32]. While the lifestyle management program is the primary service, participants are also able to access 1:1 consultations with health professionals on an as-needed basis.

To be eligible for the Logan Healthy Living lifestyle management program, participants must have a diagnosis of type 2 diabetes and be aged ≥16 years. While the program is intended for people living in the Logan region, no one is excluded from the service based on their geographical address. Participants may be excluded if it is identified by the clinical team that a group program may not be a feasible method of service delivery (ie, a participant requiring 1:1 education support). At Logan Healthy Living, services are currently delivered in English, and there is no access to free interpreter services (eg, through state or national government programs). However, clinical services have been delivered to participants who do not speak English when supported by a carer or support person.

Participants are primarily referred to the program by GPs using a referral form for group allied health services under Medicare for patients with type 2 diabetes. Participants are also able to self-refer through an expression of interest form on the service’s website. Word of mouth and marketing activities support self-referrals, such as the service’s website and newsletter, referrals from specialists (eg, the Logan Endocrine and Diabetes Service), and local advertising (eg, booths at local shopping centers, open days, and notice boards). Self-referrals may also be driven more broadly through the activities and promotion of the service by members of the alliance (eg, Metro South Health, Health and Wellbeing Queensland, Brisbane South Primary Health Network, UQ Health Care, Griffith University, and The University of Queensland). Where participants self-refer and do not have a GP referral, they are encouraged to obtain one so that the service can be reimbursed through Medicare; however, in cases in which there are barriers, this does not preclude admission into the lifestyle management program. For example, participants may experience financial barriers to primary care. While visits to GPs are subsidized by Medicare in Australia for some people, visits often attract a gap payment; services that provide no-gap primary care (bulk bill only) are limited; and some participants may not be eligible to enroll in Medicare and, therefore, ineligible for subsidized consultations. Participants can re-enroll in the program once per calendar year.

Upon presenting to Logan Healthy Living, participants are booked for an intake assessment, which is arranged over 2 appointments (appointments 1.1 and 1.2). The first appointment (1.1) is focused on medical history screening (using referral information where possible) and physical assessments. If it is identified at this first appointment that more information is required to safely prescribe exercise, the team may place the participant’s referral on hold until medical authorization to exercise is provided by the referring GP or (if self-referred) the participant’s usual GP. The second appointment (1.2) is focused on identifying barriers to participation in the lifestyle management program, goal setting, and an introduction to the exercise program. In between the 2 appointments, participants complete web-based, self-report surveys and are invited to indicate whether they agree or disagree to providing written informed consent for their data to be included in a research data registry (further details on ethics are described in the evaluation protocol in the Ethical Considerations section). Screening for completion of surveys and consent forms is conducted by the reception team, with face-to-face support for completion provided by the clinicians at the second appointment if required. After completion of the intake assessment, participants are allocated to the next available group according to their support needs and whether they are most suited for a “recently” diagnosed or “chronic” group allocation based on time since diagnosis.

### Logan Healthy Living Lifestyle Management Program

#### Overview

The lifestyle management program is the primary service delivered by Logan Healthy Living. The program was developed and is delivered in line with the American Diabetes Association Standards of Care in Diabetes [33], which outline recommendations for a range of factors related to the management of people with type 2 diabetes. In particular, the Logan Healthy Living lifestyle management program follows the key recommendations for diabetes self-management education and support, medical nutrition therapy, routine physical activity, health behavior counseling, and psychosocial care. The program incorporates a range of behavior change strategies (as described in the Behavior Change Technique Taxonomy by Michie et al [34]), including goals and planning (eg, goal setting and action planning), feedback and monitoring (eg, biofeedback), social support (eg, unspecified, emotional, and practical), shaping knowledge (eg, instruction on how to perform the behavior), comparison of behavior (eg, demonstration of the behavior), repetition and substitution (eg, graded tasks), comparison and outcomes (eg, credible source), identity (eg, framing and reframing), and self-belief (eg, verbal persuasion about capability) [34].

#### Program Structure

The lifestyle management program is delivered face-to-face in groups over 8 weeks using an interprofessional model of care. Each week includes one 2-hour session that comprises a 60-minute education workshop and a 60-minute supervised exercise (“movement”) session. Each group has a maximum of 10 participants and is assigned an education workshop and an exercise lead (either health psychologist and exercise physiologist or diabetes educator and physiotherapist, respectively) who supports and provides services to the group throughout the 8 weeks. “Recharge” sessions are offered at 1, 3, 6, 9, and 12 months following completion of the 8-week program to provide an opportunity for participants to reconnect with their health care team, review their goals, and participate in a follow-up assessment before being discharged at 12 months. The 1-month recharge is conducted face-to-face, with the remaining sessions conducted via telephone. The education workshops and supervised movement sessions are conducted on-site at Logan Healthy Living, with movement sessions conducted in the fully equipped, on-site gym.

#### Program Content

A week-by-week overview of the topics, key concepts, and resources provided in the education workshops is detailed in Table 2. The education workshops were designed to be patient centered and focus on diabetes self-management education with the clinical support needed to facilitate the knowledge, decision-making, and skill mastery necessary for diabetes self-care. Education workshop topics are integrated with social prescribing, where opportunities for community engagement related to that week’s content are signposted to support longer-term behavior change and address the broader social determinants of health. In addition to being signposted to local programs, participants are signposted to Health and Wellbeing Queensland’s suite of prevention programs, including 10,000 Steps and Deadly Choices [30]. The supervised movement session comprises an individually prescribed exercise program targeting combinations of cardiovascular fitness, strength, or flexibility depending on participant presentations. All participants are provided with the opportunity to have an individually prescribed home exercise program or access to Physitrack [35], an app that supports home exercise prescription and monitoring.

**Table 2:** Overview of the education workshop topics for the Logan Healthy Living lifestyle management program.

Week	Description	Key concepts	Handouts
1	Welcome and orientation	Acknowledgment of Country^a^ Overview of the allied health team Overview of program structure Instructions and rationale for pre-exercise checks Group introductions Group activity: “What health behaviors impact my diabetes?”	Pre-exercise checklist Lifestyle management program journey plan Opportunities for community engagement
2	Diabetes education	What is type 2 diabetes The role of insulin, insulin sensitivity, and insulin resistance Key management strategies Blood glucose testing, HbA_1c_^b^, understanding one’s “normal,” hypo- and hyperglycemia, and management strategies Effects and complications of type 2 diabetes Understanding the annual cycle of care Identifying other support (family, friends, and community) Introduction to self-management Important services to connect with	What is type 2 diabetes Key management strategies Instructions for blood glucose testing Team of support and recommended review time frames Services to find out more about type 2 diabetes—National Diabetes Services Scheme, DESMOND^c^ Australia, Diabetes Connect by Diabetes Australia, and Logan Healthy Living Facebook page
3	Nutrition part 1	Carbohydrates and how different types affect blood sugar Healthy eating guidelines Understanding what a diet looks like living with type 2 diabetes (5 main food groups) Hydration Healthy eating for diabetes Meal timing and consistency	A guide to a nourishing lifestyle—a breakdown of the 5 main food groups How to build a healthy plate Recommended meal timing Tips for staying hydrated Local services providing nutrition support
4	Nutrition part 2	Understanding food labels How food labels can help with diabetes management Grocery shopping efficiently (saving money) Modifying favorite meals to make them healthier	Tips for reading food labels Tips for enjoying home cooking and putting nutrition advice into practice Local services providing food banks
5	Movement medicine	The importance of enjoying movement what is physical activity and exercise What are the benefits of exercise The importance of exercise for type 2 diabetes Physical activity guidelines Safely exercising with type 2 diabetes Group activity: “Where can you find the motivation and inspiration to exercise?”	Benefits of exercise Pre-exercise checklist Types of movement (aerobic, balance, flexibility, and resistance training) Making exercise work for oneself Exercise and blood glucose levels Local services providing opportunities for movement
6	Stress management	Understanding stress and what happens to the body when one is stressed Understanding how stress impacts type 2 diabetes The importance of social support Group activity: managing stress—stress bucket analogy and brainstorming ways to manage stress	What is stress Identifying stressors Sources of support Local services providing well-being activities
7	Healthy habits	Understanding how to set goals effectively Review of goals and progress Planning for life after the program Creating and sustaining healthy habits Planning for future success and overcoming barriers Group activity: building healthy habits (understanding prompts, habits, and rewards) Group activity: identifying barriers and “helpers”	Tips for changing behavior and the habit cycle Ideas for goal setting related to type 2 diabetes “Planning for the future” activity—identifying what success looks like, what support systems are needed, and what has worked so far and planning for healthy habits Information on local organizations offering a range of services (eg, community centers and libraries) “Active and Healthy” booklet by the City of Logan (free and low-cost activities in the area)
8	Wrap up—review and future planning	Review of educational workshops Review of action plans (movement, community connection, nutrition, support networks, and rewards and motivations) Group activity: bingo (revision of each topic)	Summary of key takeaways from each topic How to stay connected with Logan Healthy Living (recharge session schedule, gym memberships, meet-ups, Facebook group, blogs, and allied health consultations)

^a^An Acknowledgment of Country is delivered at the beginning of each week.

^b^HbA_1c_: hemoglobin A_1c_.

^c^DESMOND: Diabetes Education and Self-Management for Ongoing and Newly Diagnosed.

### Evaluation Protocol for the Logan Healthy Living Lifestyle Management Program

Guided by the RE-AIM framework, the evaluation protocol was developed in partnership with the Health and Wellbeing Centre for Research Innovation at The University of Queensland and the other members of the steering committee and was designed to inform the service-level agreement and key questions regarding the uptake, effectiveness, costs, and sustainability of the program.

#### Study Design

The evaluation is a single-arm intervention design where participants are evaluated at intake to the service (before the program); at the end of the supervised program (8 weeks); and at approximately 1, 3, 6, 9, and 12 months after the end of the supervised program. For participants who do not re-enroll in the program, annual follow-ups are conducted at 2, 3, and 4 years.

#### Measures

##### Overview

A summary of measures that are being collected from participants to describe contextual information, program effectiveness, and acceptability is provided in Table 3, with further details and outcomes from other data sources described in the following sections. All self-report surveys are administered using REDCap, service-level data are extracted from practice management software, and physical measures are administered by clinicians on-site.

**Table 3:** Outline of participant self-report measures and timing of assessments^a^.

Measures		Structured program		Extended follow-ups				
		Intake	8 weeks	1 month	3 months	6 months	9 months	12 months
**Contextual information**								
	Sociodemographic	✓						
	Smoking and smoking changes^b^	✓	✓					✓
	Digital health use	✓	✓					✓
	Community involvement	✓	✓		✓	✓	✓	✓
**Program effectiveness**								
	Diabetes-related distress	✓	✓		✓			✓
	Health behaviors (physical activity, sitting, and nutrition)	✓	✓		✓	✓	✓	✓
	Quality of life	✓	✓		✓	✓	✓	✓
	Self-management self-efficacy	✓	✓		✓			✓
	Loneliness	✓	✓		✓			✓
	Anthropometry	✓	✓		✓	✓	✓	✓
	Physical function	✓	✓					
	HbA_1c_^c^ level	✓		✓				
	Self-reported health care use	✓						✓
**Acceptability**								
	Satisfaction		✓					

^a^All self-reported 12-month assessments (except smoking) are repeated annually at the 2-, 3-, and 4-year follow-ups.

^b^Smoking status is assessed at intake, and change in smoking status is assessed at 8 weeks and 12 months.

^c^HbA_1c_: hemoglobin A_1c_.

##### Reach Outcomes

###### Program Uptake

Program uptake will be described by reporting the number of participants who enroll in and commence the lifestyle management program compared to those considered ineligible. These outcomes will be tracked using appointment data from the practice management software, Gensolve. Withdrawals from the program and reasons for withdrawal will be tracked by clinicians using REDCap.

###### Sociodemographic and Other Contextual Characteristics

Demographic data are collected at intake on time since diagnosis of type 2 diabetes, age, sex assigned at birth, gender, postcode, First Nations status, country of birth, language spoken at home, highest level of education, occupation, and employment status. Other contextual information is collected at intake and tracked throughout the program. Smoking status is assessed at intake, and smoking changes are collected at the 8-week and 12-month follow-ups. Use of digital health technologies (eg, apps, wearables, and telehealth) is collected at intake, 8 weeks, 12 months, and then annually. Community involvement is collected at each of the assessments except for the 1-month follow-up by asking participants to report whether they take part in the following activities outside the home: social-based groups, exercise-based groups, combined social and exercise groups, and art and craft–based activities.

##### Effectiveness Outcomes

###### Diabetes-Related Distress

The primary effectiveness outcome for the evaluation is diabetes-related distress. Diabetes-related distress will be assessed using the Problem Areas in Diabetes scale [36]. The Problem Areas in Diabetes scale is a self-report, validated questionnaire that comprises 20 items assessing diabetes-related problems, with participants asked to indicate whether each item is “not a problem,” “a small problem,” “a moderate problem,” or “a somewhat serious problem.” Scores of ≥40 to 40 are considered “severe distress”; distress on specific items is considered when the total is not ≥40 but the score on one or more items is ≥3. Participants have “no evidence of distress” when both previous definitions are not met.

###### Health Behaviors

Physical activity will be assessed using the Active Australia Survey, a validated self-report measure [37]. The Active Australia Survey is designed to measure participation in leisure-time physical activity. It offers a short and reliable set of questions that can be easily administered via self-report or interview. Sitting time will be measured using an adapted version of the AusDiab multicontext sitting questionnaire, which asks participants to recall weekday and weekend day sitting time over the previous 7 days [38].

Nutrition-related behaviors will be assessed using 14 self-report items sourced from the 13-item Diet Quality Tool by O’Reilly and McCann [39] and 4 items from the evaluation of the Get Healthy Service [40], with redundant items removed. The Diet Quality Tool [39] has been validated in an Australian clinical population and reflects overall dietary quality relative to national recommendations. The New South Wales Get Healthy Service items, derived from population surveys—daily servings of fruit and vegetables as per the National Nutrition Survey [41], as well as daily servings of sweetened drinks per day and takeaway meals per week from the New South Wales Population Health Survey [42]—are useful stand-alone items for comparing results to those of other interventions such as the Get Healthy Service.

Alcohol consumption will be assessed using the brief 3-item version of the Alcohol Use Disorders Identification Test. The Alcohol Use Disorders Identification Test provides both a continuous score that correlates with alcohol consumption and adverse drinking consequences and a valid screening tool for detecting alcohol use disorders and risky drinking with validity in numerous populations, including primary care samples [43].

###### Quality of Life

Quality of life will be assessed via self-report using the EQ-5D-5L. The EQ-5D-5L is a widely used and validated tool to measure health-related quality of life [44]. The questionnaire comprises 5 dimensions—mobility, self-care, usual activities, pain or discomfort, and anxiety or depression—and participants are asked to report their level of difficulty with each dimension: *no problems*, *slight problems*, *moderate problems*, *severe problems*, and *extreme problems*. It also asks participants to self-rate their health on a numerical scale from 0 to 100.

###### Self-Management Self-Efficacy

Self-management self-efficacy will be measured via self-report using the Patient Motivation Questionnaire [45]. The Patient Motivation Questionnaire comprises 10 statements related to patients’ understanding and confidence in the self-management of their condition. The score is calculated as a total out of 10, with 8 items required to be completed.

###### Loneliness

Loneliness will be assessed via self-report using a valid and reliable scale that asks 4 questions to capture different aspects of loneliness [46]. The first 3 questions are from the University of California, Los Angeles, 3-item loneliness scale. Scores from the 3 items are used to determine whether the participants are lonely (scores of 6-9) or not lonely (scores of 3-5). The final question is a direct question on how often the respondent feels lonely.

###### Anthropometric Measures

A combination of directly measured and self-report methods will be used to assess weight (kg) and waist circumference (cm). Directly measured weight will be assessed during the 1.1 intake appointment, the 8-week group appointment, and any subsequent face-to-face recharge appointments (not including the 1-month follow-up). Where participants do not attend face-to-face follow-up appointments for direct measures, they will be asked to self-report their weight and waist circumference. Instructional videos will be provided on how participants can best self-administer these measures. The combination of self-report and direct measures is standard practice at the clinic to allow for flexibility in collecting the data in a timely way with respect to the measurement time points while also allowing participants to have measures directly taken if they are at the clinic at the time of their follow-up.

###### HbA_1c_ Measures

HbA_1c_ level will be collected using a combination of approaches. For all participants, data will be collected by state pathology laboratories, with the tests conducted closest to intake and the 1-month follow-up after the supervised program (ie, approximately 3 months after baseline) being requested from relevant data custodians. A subsample of participants will have their HbA_1c_ levels collected via finger prick at intake and at the 1-month follow-up, with the point-of-care protocol introduced in January 2024 due to availability of resources.

###### Physical Function Measures

The 2-minute step test [47], time to complete 5 sit-to-stand exercises [48], and grip strength [49] will be assessed at intake and at 8 weeks to evaluate physical function.

###### Health Care Use

Health care use will be assessed using a self-report measure of use of GP, hospital, and emergency services at baseline and the 12-month follow-up based on questions adapted from those used in the Household, Income, and Labour Dynamics in Australia Survey [50,51]. In addition, Queensland Health records will be used to quantify emergency department presentations, hospital admissions, bed days, and potentially preventable hospital admissions related to type 2 diabetes (according to the Queensland Health key performance indicator attribute sheets for diabetes-related potentially preventable hospital admissions).

##### Adoption Outcomes

Adoption will be described by estimating the number of referrals and their referral sources.

##### Implementation Outcomes

###### Fidelity

Fidelity will be assessed using adherence to the program, where adherence is the number of sessions attended. These data will be drawn from the practice management software.

###### Appropriateness

Appropriateness of the program will be assessed by continuing to monitor program adaptations. These will be tracked using a log similar to that in Table 3.

###### Acceptability

Acceptability will be determined by assessing participant satisfaction. Participant satisfaction will be assessed using a 4-point Likert scale at the end of the supervised group component of the program (8 weeks), where 1=*not at all satisfied*, 2=*somewhat satisfied*, 3=*satisfied*, and 4=*highly satisfied*. Participants will be asked to rate their overall service satisfaction, satisfaction with the quality of the services, satisfaction with the first appointment being scheduled within a reasonable period, satisfaction with the staff providing the service, and satisfaction with the written information.

###### Costs

Cost of delivery will be assessed using appointment data collected using the practice management software and overall operating costs.

##### Maintenance Outcomes

Primarily, participant maintenance will be considered using their longer-term outcomes collected at approximately 12 months after the 8-week structured program. Additional perspective will be provided by the extent of re-enrollment after 12 months (or as early as 9 months if clinically indicated), as well as the long-term outcomes at the 2-, 3-, and 4-year follow-ups among those who do not re-enroll in the program.

#### Sample Size

The number of participants receiving treatment is not connected to an a priori sample size requirement as it would be in a traditional intervention study. Nonetheless, it is useful to consider how much evaluation data provide an adequate degree of power to detect changes in outcomes. These approximate requirements, based on simple bivariate tests and presented in Table 4, show that even a brief evaluation with few participants should be adequate for some outcomes (such as physical activity), whereas a long-running evaluation with many participants might be required to detect the expected changes in sitting time or potentially clinically meaningful changes in quality of life based on the EQ-5D-5L visual analog scale.

**Table 4:** Approximate number of evaluated participants required to detect 0- to 8-week changes in effectiveness outcomes with 80% to 90% power and 5% 2-tailed significance.

Measure		Effect size	Assumed values, *r* (SD)	Required number of participants^a^	
				80% power	90% power
**Diabetes-related distress**					
	PAID-20^b^ score	MCID^c^=5	0.65 (15)	52	69
**Health behaviors**					
	Active Australia MVPA^d^ (minutes per week)	Moderate effect (0.5 SD)	0.4 1)	40	53
	Sitting time (minutes per day)	Expected effect=30	0.4 (340)	1212	1622
	Fruit consumption (servings per day)	Small effect (0.2 SD)	0.5 (1)	199	265
	Vegetable consumption (servings per day)	Small effect (0.2 SD)	0.5 (1)	199	265
	Sweet drink consumption (cups per day)	Small effect (0.2 SD)	0.5 (1)	199	265
	Takeaway meals (times per week)	Small effect (0.2 SD)	0.5 (1)	199	265
**Quality of life**					
	EQ-5D-5L visual analog scale (0-100)	MCID=6	0.6 (65)	739	989
	EQ-5D-5L index score	MCID=0.262	0.75 (0.2)	5	6
	EQ-5D-5L visual analog scale (0-100)	Small effect (0.2 SD)	0.6 (1)	159	213
	EQ-5D-5L index score	Small effect (0.2 SD)	0.75 (1)	101	134
**Self-management self-efficacy**					
	Patient Motivation Questionnaire score	Small effect (0.2 SD)	0.5 (1)	34	44
**Loneliness**					
	UCLA^e^ Loneliness Scale score	Small effect (0.2 SD)	0.75 (1)	101	134
**Anthropometry**					
	Weight (kg)	Very small change=1	0.99 (26)	427	571
	Waist circumference (cm)	Expected effect=2	0.97 (17)	36	48
**Physical function**					
	2-minute step test (steps)	MCID=11	0.65 (25)	31	40
	Sit-to-stand test (seconds)	MCID=2.3	0.65 (5.5)	34	45
	Grip strength (left hand; kg)	Expected effect=1.5 kg	0.9 (11)	87	115
	Grip strength (right hand; kg)	Expected effect=1.5 kg	0.9 (11)	87	115
**HbA_1c_^f^**					
	HbA_1c_ level (%)	MCID=0.5%	0.7 (1)	21	28
**Health care use**					
	Number of general practitioner visits	Small effect (0.2 SD)	0.5 (1)	199	265
	Number of emergency department presentations	Small effect (0.2 SD)	0.5 (1)	199	265
	Number of hospital visits	Small effect (0.2 SD)	0.5 (1)	199	265

^a^Number of participants with pre- and postevaluation data collected.

^b^PAID-20: Problem Areas in Diabetes.

^c^MCID: minimum clinically important difference.

^d^MVPA: moderate to vigorous physical activity.

^e^UCLA: University of California, Los Angeles.

^f^HbA_1c_: hemoglobin A_1c_.

#### Data Analysis

Reach, adoption, and implementation outcomes will be reported using descriptive statistics. The effectiveness of the lifestyle management program on effectiveness outcomes (all continuous) will be assessed by examining changes over time in linear mixed models accounting for repeated measures. Missing data in these mixed models will be handled through evaluable case analysis, with adjusted models including any characteristics that may differ between those providing data at different time points. The sensitivity of the conclusions to missing data handling will be evaluated using multiple imputation. All relevant time points will be reported, with the primary end point for effectiveness being 8 weeks (except for HbA_1c_ level and GP visits) and the primary end point for maintenance being approximately 12 months. The main evaluation will focus on all participants enrolled in the program, and a further per-protocol evaluation will consider outcomes for adherent participants only. Health care use in the 12 months before and the 12 months following enrolment in Logan Healthy Living will be compared using appropriate paired nonparametric tests (Wilcoxon signed ranks test and McNemar chi-square test). Costs will be estimated where possible. Sensitivity analyses will be conducted to explore whether health care use differs by a range of factors, including demographic and clinical characteristics.

### Ethical Considerations

The data registry has ethics approval from the Metro South Health Human Research Ethics Committee (project ID 84062) and has received ratification from The University of Queensland Human Research Ethics Committee (2022/HE001421). At intake, participants are provided with the participant information and consent form for the data registry by the clinical team electronically and invited to opt in by providing written informed consent. Data for the data registry are collected in an identified manner, with data stored in REDCap and The University of Queensland Research Data Manager in password-protected folders. Participants are not provided with any compensation for taking part. Hospital use data will be drawn from centrally held medical records and obtained on request from relevant data custodians.

### Iterative Adaptations to the Program

Since the opening of Logan Healthy Living in July 2021, several iterative adaptations have been made to the service delivery model to better meet the needs of the participants and the clinical team. Adaptations have been informed by stakeholder feedback (clinicians, students, and consumers) and biannual key performance indicator reports. Clinician and student reflections and feedback are discussed in regular team meetings, and participant feedback is openly encouraged through all interactions with Logan Healthy Living. Feedback from participants was sought more formally through qualitative focus groups conducted in the first years of operation that aimed to identify barriers to and facilitators of maintaining behavior change following the lifestyle management program [52]. In addition to identifying barriers that resulted in service changes (eg, ongoing access to resources such as Physitrack and the gym), participants reported that there was a sense of belonging and safety within the program, which facilitates an open dialogue for feedback on services. Key changes in response to consumer feedback are communicated to participants using a range of mechanisms, including “You said...we listened...” posters that outline feedback and corresponding changes to service delivery, which are displayed around the facility, and via clinic newsletters and social media posts. A summary of key service delivery model adaptations is provided in Table 5.

**Table 5:** Summary of key service delivery model modifications.

Year	Feedback or challenge	Modification
2022	Initially conducted as 1 appointment, the intake assessment took approximately 3 hours, and participants reported fatigue.	The intake assessment was split into 2 appointments (1.1 and 1.2).
2023	Participants wanted the opportunity to continue to attend Logan Healthy Living to exercise.	“Open” gym time was scheduled, where participants could independently use exercise equipment once they had finished the lifestyle management program.
2023	Participants had access to Physitrack during the lifestyle management program and for 3 months after, and feedback indicated that they wanted access for longer.	Participants are provided with access to Physitrack for up to 12 months after the lifestyle management program.
2024	Demand for access to the gym was increasing, and access times were unsuitable for a large number of participants.	“Open” gym concept was expanded to a full, low-cost gym membership model with expanded opening times.
2024	Recharge sessions were scheduled as a group, and attendance was poor.	To improve attendance, recharge sessions are individually scheduled and delivered face-to-face at 1 month and via telehealth (telephone) at 3, 6, 9, and 12 months.
2024	Participants expressed a desire to have ways to continue to connect socially with their peers after completion of the lifestyle management program.	A community engagement social group was started. This included a bimonthly scheduled opportunity for participants to engage with each other socially and to inform them of local community engagement activities. This activity is jointly supported by the community development team of Logan City Council.
2024	The time taken to complete the intake assessment (eg, time from referral to commencing the lifestyle management program) was resulting in high attrition and low rates of uptake of the program.	The intake process was streamlined so that appointments were booked concurrently (1.1 and 1.2) to minimize time between referral and commencing the program.
Ongoing	Feedback is continually sought on the program materials and resources from all stakeholders (participants, students, and clinicians).	Program materials and resources are iteratively adapted to respond to feedback and ensure that they are up-to-date (eg, that local services are current and still operating).

## Results

The 4-year proof of concept concludes on December 31, 2024, with reporting of the results anticipated in 2025. Results will be reported in conference abstracts and publications and with stakeholders informed through reports (with authorship determined according to scientific authorship guidelines). Findings will be shared with Logan Healthy Living participants using a range of channels, including clinic posters, newsletters, and social media.

## Discussion

### Overview

This paper describes the development and evaluation protocol for Logan Healthy Living, an interprofessional, community-delivered allied health service for people living with or at risk of type 2 diabetes in Logan, a region in South East Queensland, Australia. Delivered by a multisectoral alliance including primary and tertiary health care, government, and university partners, Logan Healthy Living is a proof-of-concept service that will be comprehensively evaluated using the RE-AIM framework and drawing from data at the level of the participants, service, and health system.

In this project, the evaluation outcomes are continuously monitored and collected in partnership between researchers and clinicians, with data collection embedded into usual practice. This is driven simultaneously by a value-based funding model and the opportunity for evidence-generating practice, with the establishment of agreed upon key service performance indicators being a pivotal influence in the buy-in required to embed this into practice. While the benefit for research is obvious, what has also emerged is the potential of continuous monitoring to inform iterative adaptations to the program, with the ability to respond in real time to optimize the health care service to suit participant and health system needs. Ongoing findings have also been used to inform collaborative strategic planning between partners to drive further development and expansion of the program and services. These “ripple effects,” such as establishing new networks, partnerships, and services to meet the needs of these adaptations, will be monitored and reported in detail in an attempt to capture the nontraditional impacts of the program on the community.

While there is a call for multisectoral responses to complex health system challenges [12], including chronic diseases such as type 2 diabetes, responses to these calls are rarely documented in detail, and the learnings from these processes are potentially lost. The detailed description of the alliance, establishment of the service, and protocol for the evaluation have the potential to inform future multisectoral partnerships and the development of community-delivered models of care for type 2 diabetes prevention and management, as well as for chronic diseases more broadly. For example, there were challenges experienced in streamlining referrals and data sharing between government health care providers and Logan Healthy Living. While they were partners within the alliance, formal pathways for these activities were not established, which meant that referrals were limited to advertising and recommendations from health professionals and participants were required to replicate intake assessments that could have been avoided, thus reducing burden. Future programs can ensure that such details are determined as part of the establishment process. The limitations of the evaluation must also be acknowledged, including the lack of a comparison group; reliance on survey data due to practical considerations, which may introduce self-report bias; and the potential high rates of missing data given that the evaluation is within a service delivery rather than research context.

### Conclusions

The implementation and evaluation of a community program within a culturally diverse region of a low socioeconomic status with rising rates of type 2 diabetes provides an opportunity to further understand the interplay of social determinants of health and chronic disease and the impact that a purposefully designed service can have on reducing the burden on the participants, the community, and the health care system. The outcomes of the evaluation will provide valuable insights into the impact of this model of care in practice, with the findings expected to inform potential scale-up through local, state, and national partnerships.

## Abbreviations

GP: general practitioner
HbA1c: hemoglobin A1c
RE-AIM: Reach, Effectiveness, Adoption, Implementation, and Maintenance
REDCap: Research Electronic Data Capture
SPIRIT: Standard Protocol Items: Recommendations for Interventional Trials

## Data Availability

Data sharing is not applicable to this paper as no datasets were generated or analyzed during this study.
